# Baicalin Protects the Cardiomyocytes from ER Stress-Induced Apoptosis: Inhibition of CHOP through Induction of Endothelial Nitric Oxide Synthase

**DOI:** 10.1371/journal.pone.0088389

**Published:** 2014-02-10

**Authors:** Mingzhi Shen, Lin Wang, Guodong Yang, Lei Gao, Bo Wang, Xiaowang Guo, Chao Zeng, Yong Xu, Liangliang Shen, Ke Cheng, Yuesheng Xia, Xiumin Li, Haichang Wang, Li Fan, Xiaoming Wang

**Affiliations:** 1 Department of Geriatrics, Xijing Hospital, Fourth Military Medical University, Xi’an, China; 2 Department of Cardiology, Xijing Hospital, Fourth Military Medical University, Xi’an, China; 3 Department of Cardiology, Hainan Branch of PLA General Hospital, Sanya, China; 4 Department of Biochemistry and Molecular Biology, Fourth Military Medical University, Xi’an, China; 5 Department of Urology, Wuhan General Hospital, Guangzhou Command, PLA, Wuhan, China; 6 Department of Geriatric Cardiology, Chinese PLA General Hospital, Beijing, China; Thomas Jefferson University, United States of America

## Abstract

Baicalin, the main active ingredient of the Scutellaria root, exerts anti-oxidant and anti-apoptotic effects in cardiovascular diseases. However, the therapeutic mechanism of baicalin remains unknown. Cultured neonatal rat cardiomyocytes were pre-treated with baicalin (0–50 µM) for 24 h, and subsequently treated with tunicamycin (100 ng/ml). Cell viability was detected by MTT assay, and cell damage was determined by LDH release and TUNEL assay. The expression of CHOP, JNK, caspase-3, eNOS was analyzed by western blot. NO was measured by DAF-FM staining. As a result, treatment with baicalin significantly reduced apoptosis induced by ER stress inducer tunicamycin in cardiomyocytes. Molecularly, baicalin ameliorated tunicamycin-induced ER stress by downregulation of CHOP. In addition, baicalin inverted tunicamycin-induced decreases of eNOS mRNA and protein levels, phospho eNOS and NO production through CHOP pathway. However, the protective effects of baicalin were significantly decreased in cardiomyocytes treated with L-NAME, which suppressed activation of nitric oxide synthase. In conclusion, our results implicate that baicalin could protect cardiomyocytes from ER stress-induced apoptosis via CHOP/eNOS/NO pathway, and suggest the therapeutic values of baicalin against ER stress-associated cardiomyocyte apoptosis.

## Introduction

The endoplasmic reticulum (ER) is recognized as an organelle that participates in the folding of secretory and membrane proteins [1], [2]. Perturbations of ER homeostasis by glucose and energy deprivation, viral infection, accumulation of unfolded and/or misfolded proteins, calcium depletion, chemical triggers such as tunicamycin, and cholesterol accumulation have been demonstrated to disrupt ER function, thus leading to ER stress [3], [4]. ER stress has been shown to participate in the pathogenesis of a wide variety of cardiovascular diseases such as ischemia reperfusion heart disease [5], [6], atherosclerosis [7], hypertension [8], myocardial infarction [9], heart failure [2], and its inhibition seems to be a promising therapeutic target. In response to ER stress, there is significant expression of ER chaperone such as glucose regulated protein 78 kD (GRP 78) [10]. When ER stress is severe and/or prolonged, however, apoptotic processes are initiated by transcriptional induction of C/EBP homologous protein (CHOP), or the phosphorylation of JNK, and/or caspase-12–dependent pathways [2].

The endothelial nitric oxide synthase (eNOS) signaling pathway plays a major role in cardioprotection. NO production from eNOS has been shown to protect cardiomyocytes from apoptosis [11], [12].

**Figure 1:**
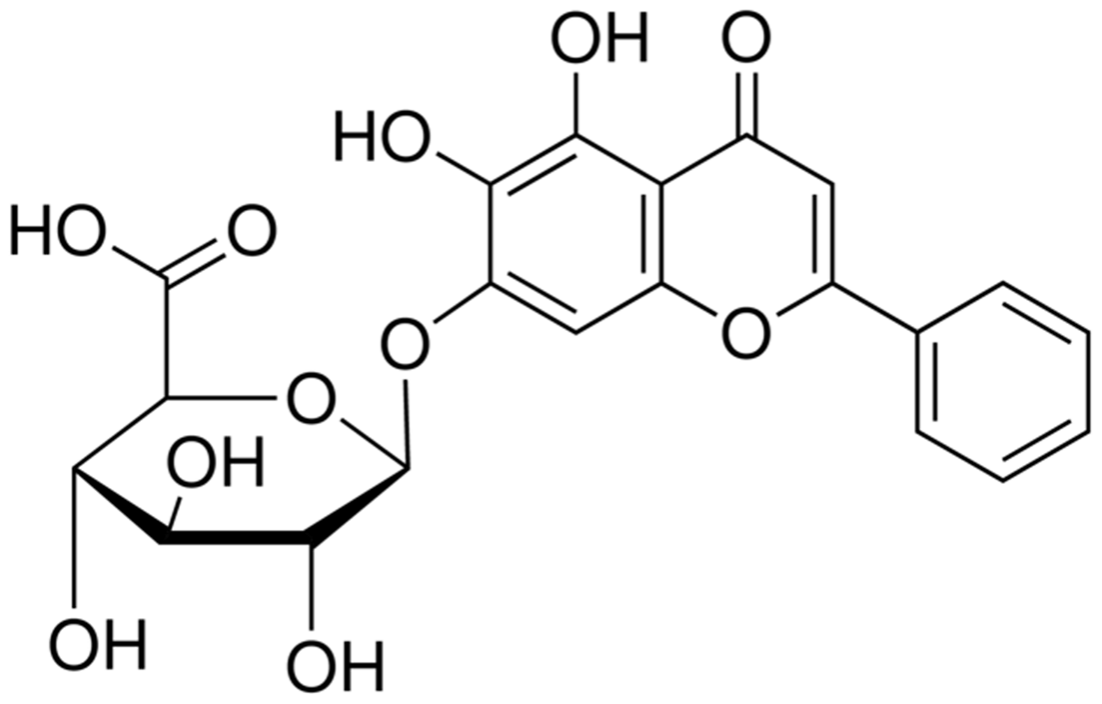
Chemical Structure of baicalin (BC).

Recently, some Chinese herbal drugs have been shown a therapeutic advantage in heart diseases. Baicalin is a flavonoid derived from the root of Scutellaria baicalensis, a commonly used Chinese herbal medicine. The chemical structure is shown in Figure 1. Baicalin exhibits anti-inflammatory, anti-oxidant, and anti-apoptotic properties [13]. However, it is still unknown whether baicalin exerts a cardioprotective effect in ER stress-induced apoptosis in cultured neonatal rat cardiomyocytes.

In this study, we hypothesized that baicalin inhibits ER stress-induced apoptosis. We further hypothesized that the anti-apoptotic effects of baicalin are mediated by increased eNOS and phospho eNOS expression, and NO production via downregulation of CHOP. To test this hypothesis, neonatal cardiomyocytes were cultured, and apoptosis was stimulated by ER stress inducer tunicamycin. Our data showed that baicalin attenuated ER stress-induced apoptosis. Furthermore, the cardioprotective effect induced by baicalin is at least partially due to inhibition of CHOP and subsequent eNOS, phospho eNOS and NO upregulation. Taken together, these findings suggest that baicalin might be a promising therapeutic agent for the treatment of ER stress-mediated cardiovascular diseases.

## Materials and Methods

### Reagents and Antibodies

Tunicamycin, baicalin, 5-bromo-2-deoxyuridine (BrdU), 3-(4,5-dimethylthiazol-2-yl)-2,5-diphenyltetrazolium bromide (MTT), Lactate dehydrogenase (LDH), L-NAME (NOS inhibitor), and antibody for β-actin were purchased from Sigma-Aldrich Corporation (St. Louis, MO, USA). Antibody against GRP 78 was from Bioworld Technology (St. Louis Park, MN, USA). Antibodies against CHOP and eNOS were from Santa Cruz Biotechnology (Santa Cruz, CA, USA). Antibodies against caspase-3 and phospho eNOS were from Cell Signaling Technology (Danvers, MA, USA). DAF-FM was from Molecular Probes (Eugene, OR, USA). Terminal Deoxynucleotidyltransferase-mediated dUTP Nick End Labeling (TUNEL) was from Roche Applied Science (Sandhofer Strasse, Mannheim, Deutschland).

### Primary Culture of Cardiomyocytes

Cardiomyocytes were prepared from newborn Sprague–Dawley rats as described previously [12]. In brief, neonatal rat ventricles were enzymatically digested, and cardiomyocytes were purified through 1 h incubation (37°C in a 5% CO_2_ incubator). After that, cardiomyocytes were cultured in DMEM medium containing 10% fetal bovine serum and 100 µM BrdU for 16–24 hours. The yield of cardiomyocytes was over 90% as determined by α-actinin staining method. All procedures involving animals were in accordance with the Guide for the Care and Use of Laboratory Animals published by the US National Institutes of Health (NIH Publication No.85-23, revised 1996), and approved by the Fourth Military Medical University Committee on Animal Care.

### Determination of Cell Viability

Cell viability was assessed by the MTT assay as described previously with minor modifications [2]. Briefly, Cells were seeded into 96-well culture plates at a density of 5×10^4^/well (100 µl). After treatment, 10 µl MTT solution (5 mg/ml in PBS) was added into each well and incubated at 37°C for 4 h. Then the medium was removed, and 150 µl DMSO was added into each well. The plate was gently shaken for 10 min to completely dissolve the precipitation. The absorbance was detected at 490 nm (Bio-Rad, USA).

### Measurement of Lactate Dehydrogenase (LDH)

To determine cardiomyocyte damage, LDH release in medium was detected using an LDH kit according to the manufacturer’s instructions. The LDH release was shown as a rate of LDH released in the medium to total cellular LDH.

### TUNEL Assay

The in situ TUNEL cell death detection kit was used to detect apoptotic cells according to manufacturer’s instructions. Briefly, cells were fixed with 4% paraformaldehyde and permeabilized by 0.3% Triton X-100, and then washed twice with PBS. DNA breaks were labeled by incubation (1 h, 37°C) with terminal deoxynucleotidyltransferase and nucleotide mixture containing fluorescein isothiocyanate-conjugated dUTP. Cells were nuclear stained with DAPI, and the TUNEL positive and total nuclei were observed under a laser scanning confocal microscope (Nikon, Japan).

### Western Blot Analysis

Cardiomyocytes were lysed with RIPA containing a protease inhibitor cocktail (Roche). Electrophoresis and immunoblotting were done as described previously [14]. For the densitometry analysis, optical density was measured on the inverted digital images using Image J software.

### Real-time Quantitative Polymerase Chain Reaction (PCR)

cDNA synthesis was performed with QuantiTect Reverse Transcription Kit (TaKaRa Biotech. Co. Ltd., China). PCR was performed on an ABI prism 7500 with the Power SYBR Green PCR Master Mix (TaKaRa Biotech. Co. Ltd., China). Rat β-actin was used to normalize sample amplification. The following primer sequences were used: eNOS forward: 5′-CTACCGGGACGAGGTACTGG-3′ and reverse: 5′-GGAAAAGGCGGTGAGGACTT-3′; β-actin forward: 5′-AGAGGGAAATCGTG CGTGAC-3′ and reverse: 5′-TTCTCCAGGGAGGAAGAGGAT-3′.

### RNA Interference

CHOP–specific short interfering RNA (siRNA) was purchased from GenePharma. Target sequence of siRNA was 5′-CGAAGAGGAAGAAUCAAA-3′, and the sequence of negative control was 5′-UUCUCCGAACGUGUCACGUTT-3′. Transfection was performed as described previously [15].

### NO Production

NO production in cardiomyocytes was measured using 4-amino-5-methylamino-2′,7′-difluorofluorescein (DAF-FM, Molecular Probes). Briefly, cardiomyocytes were cultivated as described above but cultured in 96-well black plates. Cells were incubated with 10 µM DAF-FM for 2 hours (37°C). Supernatants were then removed, and cardiomyocytes were washed with DAF-FM-free buffer. The fluorescence intensity generated from NO was detected using a fluorescent microplate reader (Wallac) with excitation and emission wavelengths of 495 nm and 515 nm, respectively. Results are presented as the percentage of control.

### Statistical Analysis

All experiments were performed five times. Results were expressed as mean ± SEM. All data were subjected to ANOVA, followed by Bonferroni correction for post-test. A value of P<0.05 was considered statistically significant.

## Results

### ER-stress Related Cell Injury in Tunicamycin-treated Cardiomyocytes

**Figure 2:**
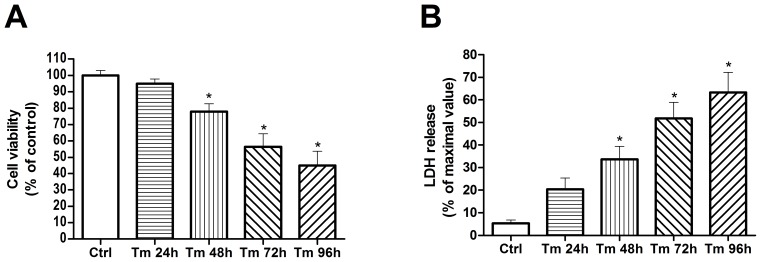
Cardiomyocyte viability upon tunicamycin treatment. Cardiomyocytes were treated with 100/ml tunicamycin (Tm) for indicated time (0, 24 h, 48 h, 72 h, 96 h) and cell viability was measured by the MTT-assay (A) and cell injury tested by the LDH-assay (B). For cell viability, the viable cell number was compared with Control group. For the LDH release assay, all the values were compared with Control group. *P<0.05 vs Ctrl, n = 5.

MTT assay and LDH release assay in tunicamycin-treated cardiomyocytes were used to evaluate cell injury. 100 ng/ml tunicamycin was selected to induce ER stress as described previously [2]. Compared with Control group, tunicamycin for 24 h had no effect on cell viability or LDH release, whereas cell viability decreased, and LDH release increased after 48- to 96-h exposure to tunicamycin (Figure 2A and 2B). In addition, tunicamycin led to significant cell injury in cardiomyocytes in a time-dependent manner.

### Baicalin Attenuated Tunicamycin-induced Injury in Cardiomyocytes

**Figure 3:**
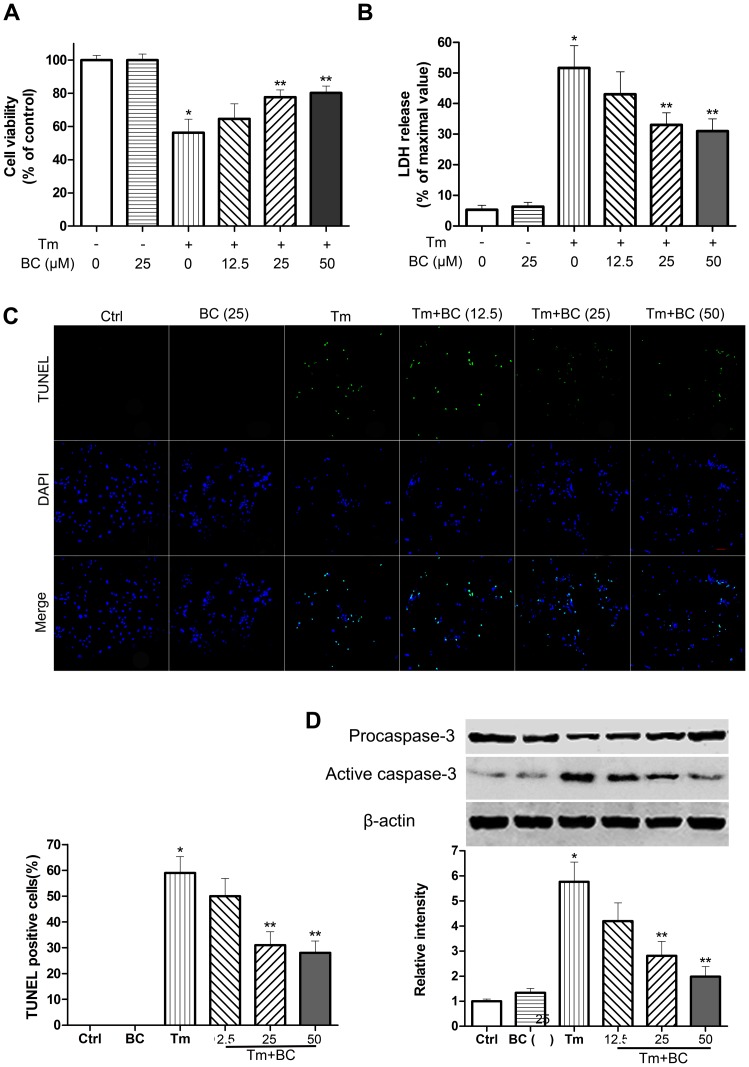
Protective effect of baicalin on tunicamycin-induced cardiomyocyte death. Cardiomyocytes were treated with Tm (100 ng/ml) in the presence or absence of baicalin (BC) at indicated concentrations (12.5, 25, 50 µM). (A) Protective effect of baicalin (BC, 72 h) on Tm-induced cardiomyocyte viability measured by MTT-assay. (B) Protective effect of baicalin (BC, 72 h) on Tm-induced cardiomyocyte injury measured by the LDH-assay. (C) Cardiomyocytes were treated same as above and cell apoptosis was detected by TUNEL assay. Quantitative analysis of TUNEL-positive cardiomyocytes after Tm treatment with or without BC for 72 h. (D) The expression of caspase-3 in cardiomyocytes treated same as above was tested by western blot. *P<0.05 vs Ctrl, **P<0.05 vs Tm, n = 5.

To investigate whether baicalin could protect cardiomyocytes from tunicamycin-induced injury, cardiomyocytes were pretreated with baicalin at different concentrations (0, 12.5, 25, 50 µM) for 24 h before exposure to tunicamycin for 72 h. When cardiomyocytes were treated with baicalin (25 µM) alone, there were no obvious effect on survival cell number (Figure 3A), LDH release (Figure 3B). Compared with Control group, 12.5 µM baicalin had a mild but not significant prosurvival role, as seen from MTT assay (Figure 3A), LDH release (Figure 3B), apoptosis (Figure 3C), or cleavage of caspase-3 (Figure 3D); whereas pretreatment with higher dose of baicalin (25 or 50 µM) improved survival number (Figure 3A), and reduced LDH leakage (Figure 3B), apoptosis (Figure 3C), and cleavage of caspase-3 (Figure 3D).

### Baicalin Ameliorated Tunicamycin-induced CHOP Expression in Cardiomyocytes

**Figure 4:**
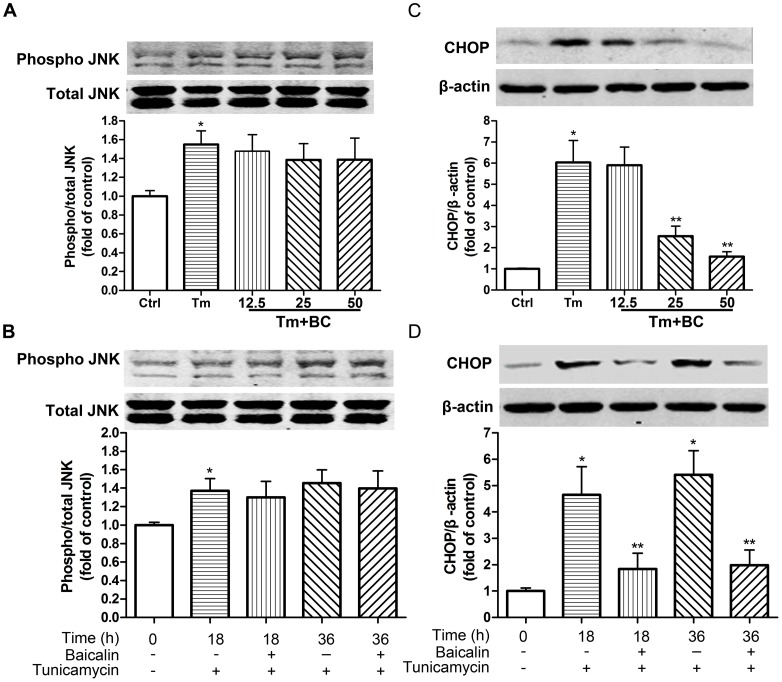
Baicalin reduced Tm-induced CHOP expression in cardiomyocytes. (A, C) Cardiomyocytes were treated with Tm (100 ng/ml) in the presence or absence of baicalin (BC) for 24 h at indicated concentrations (12.5, 25, 50 µM). (B, D) Cardiomyocytes were treated with Tm (100 ng/ml) in the presence or absence of baicalin (BC,25 µM) for 24 h at indicated times (0, 18 h, 36 h). (A, B) Representative immunoblotting and quantitative assay of phosphorylation of JNK protein expressions. (C, D) Western blot analysis and quantitative assay of CHOP protein expression. β-actin or total JNK served as loading control. *P<0.05 vs Ctrl, **P<0.05 vs Tm, n = 5.

To explore the underlying protective mechanism of baicalin on tunicamycin- induced injury, we examined the common signaling pathways of ER-initiated apoptosis in cardiomyocytes. As expected, tunicamycin increased the expression of phospho JNK. While there were little effect of baicalin on tunicamycin-induced upregulation of phospho JNK (Figure 4A and 4B). These data suggested that the protective effect of baicalin on tunicamycin-induced injury was independent of phospho JNK. We further tested the effect of baicalin on the expression of CHOP in cardiomyocytes. Pretreatment with baicalin markedly attenuated tunicamycin-induced upregulation of CHOP (Figure 4C and 4D).

### Baicalin Inverted Tunicamycin-induced eNOS through CHOP–dependent Regulation

**Figure 5:**
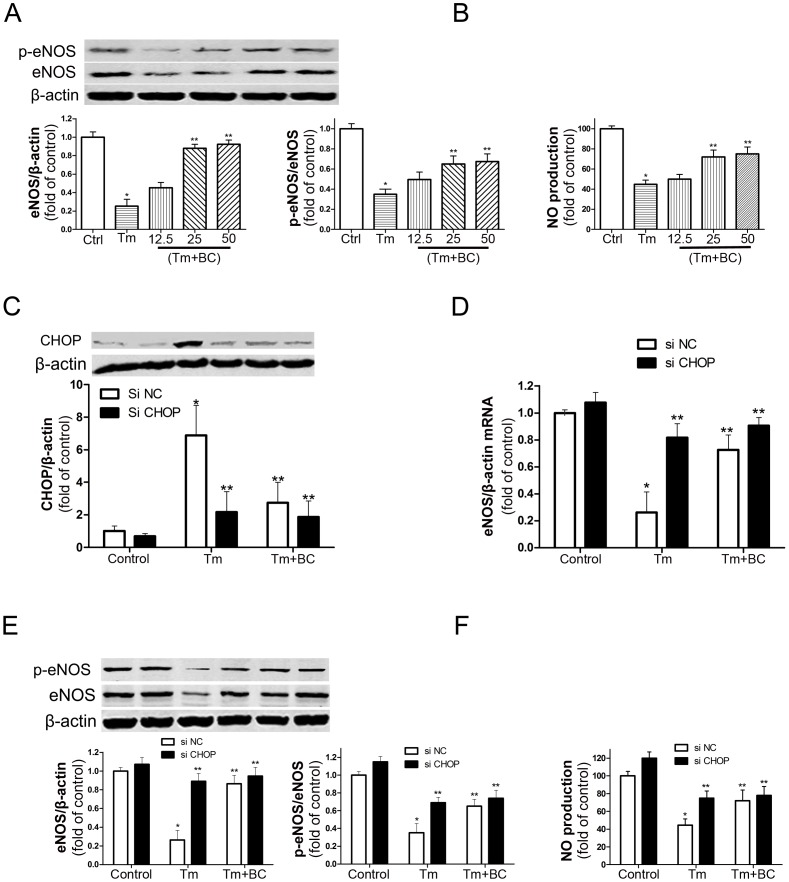
CHOP dependent induction of endothelial nitric oxide synthase (eNOS) in BC treated cardiomyocytes. (A) Cardiomyocytes were treated with Tm (100 ng/ml) in the presence or absence of baicalin (BC) for 24 h at indicated concentrations (12.5, 25, 50 µM). The expressions of eNOS and phospho eNOS at protein levels were examined by western blot. (B) BC treatment increased NO production in cultured cardiomyocytes. Cardiomyocytes were treated with Tm (100 ng/ml) in the presence or absence of baicalin (BC) for 24 h and incubated with 10 µM of DAF-FM and fluorescence intensity was assayed. (C) CHOP knockdown efficiency was tested by western blot. Cells transfected with either control siRNA or siRNA targeting CHOP were further treated with Tm (100 ng/ml) in the presence or absence of baicalin (BC, 25 µM) for 24 h. (D) The expression of eNOS mRNA levels was examined by real-time quantitative PCR. Cells were treated as C. (E) The expressions of eNOS and phospho eNOS protein levels were examined by western blot. Cells were treated as C. (F) NO production was measured by DAF-FM and fluorescence intensity was assayed. Cells were treated as C. A, B: *P<0.05 vs Ctrl, **P<0.05 vs Tm, n = 5; C, D, E, F: *P<0.05 vs Control+si NC, **P<0.05 vs Tm+si NC, n = 5.

To explore the involvement of eNOS on ER stress inducer tunicamycin-induced cardiomyocyte injury, eNOS and NO were examined in cardiomyocytes. Our data showed that tunicamycin downregulated eNOS, phospho eNOS and NO expressions (Figure 5A and 5B). Upon treatment of tunicamycin (100 ng/ml, 24 h), expressions of eNOS, phospho eNOS and NO reversely correlated with CHOP (Figure 5). Interestingly, si CHOP prevented the downregulation of eNOS mRNA and protein levels, phospho eNOS and NO induced by tunicamycin (Figure 5E and 5F), suggesting that eNOS is a CHOP target in vitro. Pretreatment with baicalin markedly increased eNOS, phospho eNOS proteins and NO level compared with tunicamycin group. Whereas si CHOP and baicalin combination had no extra effect on eNOS mRNA and protein levels, phospho eNOS or NO production, compared with si CHOP or baicalin alone. Taken together, baicalin inverts tunicamycin-induced eNOS expression through CHOP–dependent regulation.

### Baicalin Protected Cardiomyocytes through CHOP–eNOS Pathway

**Figure 6:**
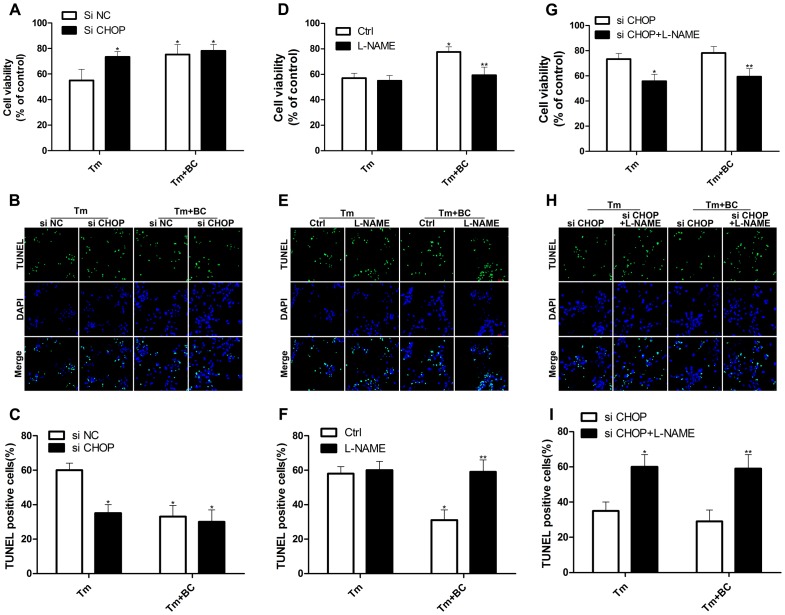
CHOP-eNOS pathway in baicalin mediated protective role. Cardiomyocytes were transfected with or without siRNA for 24(BC, 25 µM) and L-NAME (10 µM) pretreatment. Viability was measured by the MTT-assay in cardiomyocytes (A, D, G). Apoptosis was tested by TUNEL analysis (B, E, H). Quantitative analysis of TUNEL-positive cardiomyocytes (C, F, I). A, B, C: *P<0.05 vs Tm+si NC, n = 5; D, E, F: *P<0.05 vs Ctrl+Tm, **P<0.05 vs Ctrl+Tm+BC, n = 5; G, H, I: *P<0.05 vs Tm+si CHOP, **P<0.05 vs Tm+BC+si CHOP, n = 5.

To examine the role of CHOP, si CHOP was used to suppress CHOP. si CHOP and/or pretreatment of baicalin improved the cell survival (Figure 6A, 6B and 6C). Whereas si CHOP and baicalin together had no extra effect on cell death induced by tunicamycin, compared with si CHOP or baicalin alone. Taken together, baicalin protects cardiomyocytes from tunicamycin-induced apoptosis through CHOP. To investigate the role of eNOS in baicalin-induced protective effect, L-NAME was used to inhibit eNOS. L-NAME itself had no obvious effect on cell apoptosis (data not shown). In contrast, the anti-apoptotic effect of baicalin was nearly fully blunted by L-NAME (Figure 6D, 6E, and 6F). Taken together, baicalin protects cardiomyocytes from tunicamycin-induced apoptosis through regulation of eNOS. To determine the functional significance of the CHOP-eNOS pathway, si CHOP and L-NAME were used to inhibit CHOP and eNOS, respectively. The anti-apoptotic effect of si CHOP and/or baicalin was nearly inhibited by L-NAME (Figure 6G, 6H, and 6I). Taken together, these results support the concept that baicalin protects cardiomyocytes from tunicamycin-induced apoptosis through CHOP–dependent regulation of eNOS expression.

## Discussion

The root of Scutellaria baicalensis has been widely used in traditional Chinese medicine for thousands of years to treat various diseases, including cardiovascular diseases [16]. Baicalin, a main active ingredient originally isolated from the root of Scutellaria baicalensis, possesses multiple biochemical and pharmacological activities including anti-oxidant, anti-inflammatory, anti-apoptosis, anti-bacterial, immune-stimulating, anti-allergic, and anti-viral effects [17], [18]. However, its role in the regulation of ER stress-induced apoptosis in cardiomyocytes has not been elucidated. This study for the first time examined the effects of baicalin on ER stress-induced apoptosis in cardiomyocytes. In this study, tunicamycin was used to induce ER stress. We found that baicalin attenuated tunicamycin-induced cardiomyocyte injury.

ER stress-induced apoptosis has been implicated in the pathophysiology of various human diseases, including cardiovascular diseases such as cardiac hypertrophy, ischemia/reperfusion injury of the heart, ischemic heart diseases, atherosclerosis, alcoholic cardiomyopathy, autoimmune cardiomyopathy [19], myocardial infarct, and heart failure [20], [21], [22]. Tunicamycin is a specific inhibitor of N-linked glycosylation, which is observed only in the ER, showing that tunicamycin is a highly specific ER stress inducer [2]. Our data reveal that tunicamycin promotes LDH release and reduces cell viability, leading to significant cardiomyocyte injury in a time-dependent manner.

Although baicalin has been shown to be protective in other systems [23], [24], cell death is often regulated in a type- and stimulus-specific manner. Therefore, we studied the role of baicalin in ER stress-induced apoptosis. In our culture system, cytotoxic effect of baicalin was not observed at 12.5, 25, and 50 µM in the absence of tunicamycin. Our study revealed, for the first time, that pretreatment with different concentrations of baicalin exhibits cardioprotective activity against ER stress-induced apoptosis by tunicamycin (improved cell survival, and reduced LDH release and apoptosis) in a supplied dose-dependent manner. Therefore, it is conceivable that baicalin could be used to treat ER stress-induced apoptotic diseases, including cardiovascular diseases.

However, the mechanism underlying the anti-apoptotic effect of baicalin remains unclear. ER stress induces apoptotic processes through transcriptional induction of CHOP, activation of JNK, and caspase-12–dependent signaling pathways [2]. We demonstrated that tunicamycin activated JNK, and significantly increased CHOP levels. JNK is important in ER stress-induced apoptosis; however, cardioprotective effect of baicalin is unlikely by mediating JNK, because pretreatment with baicalin had little effect on their expressions. Pretreatment with baicalin significantly decreased tunicamycin-induced CHOP protein level in a concentration-dependent manner. These results suggest that baicalin attenuates tunicamycin-induced ER stress through CHOP pathway. However, the precise downstream of CHOP remains to be further clarified.

Endothelial nitric oxide synthase (eNOS) is an essential cell protective mediator under multiple conditions [11], [25]. eNOS is an important downstream effector in the anti-apoptotic signaling by insulin in MI/R [26], [27]. Adenovirus mediated human eNOS gene therapy administered in animals four days before MI leads to lesser apoptosis [28]. Neuronal nitric oxide synthase protects the pancreatic beta cells from glucolipotoxicity-induced endoplasmic reticulum stress and apoptosis [29]. Endoplasmic reticulum stress-mediated inhibition of NSMase2 elevates plasma membrane cholesterol and attenuates NO production in endothelial cells [30]. eNOS is negatively regulated by CHOP-10 in the process of postnatal neovascularization [31]. However, if eNOS participates in ER stress-induced cardiomyocyte apoptosis has not been reported. In our present study, tunicamycin downregulated eNOS mRNA and protein levels, phospho eNOS, and NO production; while si CHOP inverted eNOS mRNA and protein levels, phospho eNOS, and NO level induced by tunicamycin. These indicate that eNOS is a CHOP–regulated gene in cardiomyocytes. Pretreatment with baicalin significantly increased eNOS, phospho eNOS levels, and NO production; however, si CHOP and baicalin had no extra effects on eNOS, phospho eNOS levels, and NO production induced by tunicamycin. These results demonstrate that baicalin reveres tunicamycin-induced eNOS expression through CHOP–dependent pathway.

In conclusion, our study suggests a protective effect of baicalin on tunicamycin-induced cardiomyocyte apoptosis, which is mediated by CHOP repression and subsequent eNOS, phospho eNOS and NO induction. Our study here indicates that baicalin might be a promising therapeutic agent for the treatment of cardiovascular diseases.
